# Defining the Molecular Basis for the First Potent and Selective Orthosteric Agonists of the FFA2 Free Fatty Acid Receptor*

**DOI:** 10.1074/jbc.M113.455337

**Published:** 2013-04-15

**Authors:** Brian D. Hudson, Maria E. Due-Hansen, Elisabeth Christiansen, Anna Mette Hansen, Amanda E. Mackenzie, Hannah Murdoch, Sunil K. Pandey, Richard J. Ward, Rudi Marquez, Irina G. Tikhonova, Trond Ulven, Graeme Milligan

**Affiliations:** From the ‡ Molecular Pharmacology Group, Institute of Molecular, Cell, and Systems Biology, College of Medical, Veterinary, and Life Sciences, Wolfson Link Building 253, University of Glasgow, Glasgow G12 8QQ, United Kingdom,; the § Department of Physics, Chemistry, and Pharmacy, University of Southern Denmark, Campusvej 55, DK-5230 Odense M, Denmark,; the ¶ School of Chemistry, University of Glasgow, Glasgow G12 8QQ, United Kingdom, and; the ‖ School of Pharmacy, Medical Biology Centre, Queen's University Belfast, Belfast BT9 7BL, United Kingdom

**Keywords:** 7-Helix Receptor, Adipocyte, Fatty Acid, G Protein-coupled Receptors (GPCRs), Signal Transduction

## Abstract

FFA2 is a G protein-coupled receptor that responds to short chain fatty acids and has generated interest as a therapeutic target for metabolic and inflammatory conditions. However, definition of its functions has been slowed by a dearth of selective ligands that can distinguish it from the closely related FFA3. At present, the only selective ligands described for FFA2 suffer from poor potency, altered signaling due to allosteric modes of action, or a lack of function at non-human orthologs of the receptor. To address the need for novel selective ligands, we synthesized two compounds potentially having FFA2 activity and examined the molecular basis of their function. These compounds were confirmed to be potent and selective orthosteric FFA2 agonists. A combination of ligand structure-activity relationship, pharmacological analysis, homology modeling, species ortholog comparisons, and mutagenesis studies were then employed to define the molecular basis of selectivity and function of these ligands. From this, we identified key residues within both extracellular loop 2 and the transmembrane domain regions of FFA2 critical for ligand function. One of these ligands was active with reasonable potency at rodent orthologs of FFA2 and demonstrated the role of FFA2 in inhibition of lipolysis and glucagon-like peptide-1 secretion in murine-derived 3T3-L1 and STC-1 cell lines, respectively. Together, these findings describe the first potent and selective FFA2 orthosteric agonists and demonstrate key aspects of ligand interaction within the binding site of FFA2 that will be invaluable in future ligand development at this receptor.

## Teaser

**Background:** Understanding the function of FFA2 has been slowed by a lack of selective orthosteric ligands.

**Results:** Residues within FFA2 that dictate the recognition and function of potent and selective orthosteric agonists are described.

**Conclusion:** Key aspects of ligand interaction with the orthosteric binding pocket of FFA2 are defined.

**Significance:** This work will be invaluable in future drug development at the FFA2 receptor.

## Introduction

G protein-coupled receptors (GPCRs)^2^ activated by free fatty acids have generated significant interest in recent years for their potential use as therapeutic targets in the treatment of various metabolic and inflammatory conditions. These GPCRs include a family of four receptors: two that respond to long chain fatty acids (FFA1 (previously GPR40) and FFA4 (previously GPR120)) and two that respond to short chain fatty acids (SCFAs) (FFA2 (previously GPR43) and FFA3 (previously GPR41)). Although the long chain fatty acid receptors have received the most interest, FFA1 due to its ability to potentiate glucose-stimulated insulin release (1) and FFA4 for its ability to stimulate glucagon-like peptide-1 secretion (2) and to mediate the insulin-sensitizing and anti-inflammatory properties of *n*-3 fatty acids (3), the SCFA receptors, and particularly FFA2, have also generated significant interest. However, the development and validation of FFA2 as a therapeutic target has been hindered greatly by a lack of suitably selective ligands for this receptor over the closely related FFA3 (4).

Although both FFA2 and FFA3 respond to the same group of SCFA ligands, they display clear differences in the rank order of potency for these ligands. Of particular interest, human FFA2 (hFFA2) responds with equal potency to acetate (C2) and propionate (C3), whereas hFFA3 displays greater potency for C3 than for C2 (5–7). Although this observation has led to some use of C2 as a selective agonist for FFA2 (8, 9), both the potency and selectivity for C2 at hFFA2 remain low (10), making it impractical to use this ligand effectively to differentiate clearly between FFA2 and FFA3 function. More importantly, we have recently demonstrated that although the rank order difference is maintained, the selectivity for C2 is not preserved in the rodent orthologs of FFA2 and FFA3, where C2 was found to be equipotent at mouse FFA2 (mFFA2) and mFFA3 (11). Clearly, this demonstrates a need for synthetic ligands that can differentiate between the function of these receptors in both human and rodent systems.

Early attempts to identify synthetic ligands for FFA2 revealed a series of phenylacetamides, exemplified by 4-chloro-α-(1-methylethyl)-*N*-2-thiazolylbenzeneacetamide (4-CMTB), as the first reasonably potent and selective agonists of this receptor (12). However, detailed examination of the pharmacology of these compounds has demonstrated that they are in fact ago-allosteric modulators of FFA2, binding to a site distinct from the endogenous SCFAs (12, 13). Although 4-CMTB is active at rodent orthologs of FFA2 (11) and has been used to define some aspects of FFA2 function (12, 14), it is also apparent that as an allosteric modulator, signaling responses to 4-CMTB at FFA2 are not identical to those of the endogenous SCFAs (13). Considering this, there is clearly a need to develop novel FFA2 selective ligands with properties more similar to, and which ideally bind to the same site as, the endogenous SCFAs. To date, the only study describing selective orthosteric agonists for FFA2 examined the structure-activity relationship of small carboxylic acids to identify a number of compounds with modest selectivity for both FFA2 and FFA3 (10). However, due to their relatively small size and resulting low binding energy, the potency of these small carboxylic acid agonists remains low.

Selective antagonists for the SCFA receptors would be equally useful in helping to define the specific functions of FFA2 compared with FFA3, and, indeed, at least one FFA2 antagonist/inverse agonist, (*S*)-3-(2-(3-chlorophenyl)acetamido)-4-(4-(trifluoromethyl)phenyl) butanoic acid (CATPB), has been described (15). However, the practical utility of this compound is reduced substantially by the fact that it appears to be highly selective for the human ortholog, with no significant affinity at mFFA2 (11). Given the lack of potent and selective orthosteric agonists or antagonists with activity in non-human systems, there is clearly a substantial need for novel FFA2 ligands if the function of this receptor is to be fully explored and understood.

To address this need, we identified compounds from the patent literature with reported activity at FFA2 that we hypothesized were likely to be orthosteric agonists, based primarily on the presence, as in the SCFAs, of a carboxylate moiety (16). We synthesized two compounds based on the structures described and examined their function at human and rodent orthologs of the FFA receptors. This confirmed these compounds to be potent and selective orthosteric FFA2 agonists. We then used a combination of ligand-based structure activity, detailed pharmacological and functional analysis, homology modeling, species ortholog comparisons, and mutational analysis to define key molecular determinants of how these ligands interact with FFA2 and then used the ligand with similar potency at human and mouse FFA2 to define the role of this receptor in both the regulation of lipolysis in model adipocytes and in the release of the incretin glucagon-like peptide-1 (GLP-1) from enteroendocrine cells.

## EXPERIMENTAL PROCEDURES

### 

#### 

##### Materials

**FIGURE 1.**
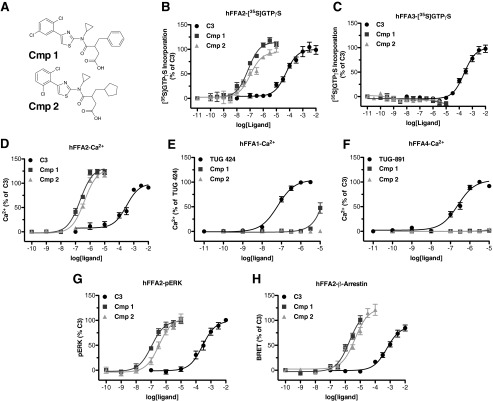
**Compounds 1 and 2 are potent and selective agonists of FFA2.** Chemical structures of compounds **1** and **2** are shown in *A*. Concentration-response curves for **C3**, **1**, and **2** across various function assays are shown for hFFA2 (*B*, *D*, *G*, and *H*), hFFA3 (*C*), hFFA1 (*E*), and hFFA4 (*F*). Assays chosen represent [^35^S]GTPγS to assess G_i/o_ coupling (*B* and *C*), Ca^2+^ mobilization to assess G_q/11_ signaling (*D–F*), ERK phosphorylation (*G*), and β-arrestin-2 recruitment (*H*). *Error bars*, S.E.

FFA2 ligands were synthesized based on the methods described previously (16). The identity of each compound was confirmed by NMR spectroscopy. Synthesized compound, compound **1** (see Fig. 1), was 3-benzyl-4-(cyclopropyl-(4-(2,5-dichlorophenyl)thiazol-2-yl)amino)-4-oxobutanoic acid. The purity of this compound was determined by HPLC analysis performed using a Dionex 120 C18 column (5 μm, 4.6 × 150 mm) with 10% acetonitrile in water (0–1 min), 10–100% acetonitrile in water (1–10 min), 100% acetonitrile (11–15 min), both solvents containing 0.05% TFA as modifier, a flow of 1 ml/min, and UV detection at 230 and 254 nm. *Rt* = 15.9 min, >99% pure. ^1^H NMR (400 MHz, CDCl_3_) δ 11.64 (br s, 1H), 7.96 (d, *J* = 2.6 Hz, 1H), 7.62 (s, 1H), 7.41–7.10 (m, 7H), 4.18 (s, 1H), 3.14–2.85 (m, 4H), 2.56–2.48 (m, 1H), 1.26–1.22 (m, 4H); ^13^C NMR (101 MHz, CDCl_3_) δ 177.2, 176.7, 137.7, 134.7, 132.8, 131.6, 131.1, 130.2, 129.2, 128.98, 128.95, 128.8, 128.5, 127.7, 127.1, 41.4, 38.7, 35.6, 33.0, 11.4; ESI-HRMS calculated for C_23_H_20_Cl_2_N_2_O_3_S (M + H^+^) 475.0641, found 475.0644). The ClogP of this compound was calculated to be 5.2 using the ChemBioDraw software package. Compound **2** is (*R*)-3-(cyclopentylmethyl)-4-(cyclopropyl-(4-(2,6-dichlorophenyl)thiazol-2-yl)amino)-4-oxobutanoic acid. ^1^H NMR (400 MHz, CDCl_3_) δ (ppm): 7.38 (1H, d, *J* = 8.1 Hz), 7.24–7.22 (2H, m), 7.05 (1H, br s), 3.23–3.17 (1H, m), 2.95 (1H, dd, *J* = 16.6, 10.5 Hz), 2.65 (1H, dd, *J* = 16.6, 4.3 Hz), 1.92–1.74 (3H, m), 1.71–1.49 (5H, m), 1.34–1.19 (4H, m), 1.18–1.05 (2H, m), 1.00–0.90 (2H, m). A ClogP of 5.4 was calculated for compound **2**. The methyl ester of compound **2** was (*R*)-3(cyclopentylmethyl)-4-(cyclopropyl(4-(2,6-dichlorophenyl)thiazol-2-yl)amino)-4-oxobutanoic acid methyl ester; ^1^H NMR (400 MHz, CDCl_3_) δ (ppm): 7.40–7.39 (1H, m), 7.38–7.37 (1H, m), 7.24–7.21 (1H, m), 7.03 (1H, br s), 4.02–3.94 (1H, m), 3.68 (3H, s), 3.23–3.18 (1H, m), 2.93 (1H, dd, *J* = 17.1, 10.0 Hz), 2.61 (1H, dd, *J* = 17.1, 5.2 Hz), 1.90–1.73 (4H, m), 1.67–1.47 (5H, m), 1.34–1.18 (3H, m), 1.17–1.06 (2H, m), 1.02–0.95 (1H, m). The *tert*-butyl ester ester of compound **2** was (*R*)-3(cyclopentylmethyl)-4-(cyclopropyl(4-(2,6-dichlorophenyl)thiazol-2-yl)amino)-4-oxobutanoic acid *tert*-butyl ester; ^1^H NMR (400 MHz, CDCl_3_) δ (ppm): 7.42 (1H, d, *J* = 8.1 Hz), 7.26–7.24 (2H, m), 7.06 (1H, br s), 3.27–3.21 (1H, m), 2.97 (1H, dd, *J* = 17.0, 10.0 Hz), 2.65 (1H, dd, *J* = 17.0, 4.3 Hz), 1.95–1.76 (3H, m), 1.74–1.54 (3H, m), 1.62 (9H, s), 1.44–1.21 (5H, m), 1.20–1.10 (3H, m), 1.06–0.90 (2H, m).

4-CMTB was synthesized as described previously (13). The hFFA2 antagonist/inverse agonist CATPB was synthesized as described previously (11). The FFA4 agonist, 3-(4-((4-fluoro-4-methyl-[1,1-biphenyl]-2-yl)methoxy)phenyl)propanoic acid (TUG-891), was synthesized as described by Shimpukade *et al.* (17). Tissue culture reagents were from Invitrogen. Molecular biology enzymes and reagents were from Promega (Southampton, UK). The radiochemical [^35^S]GTPγS was from PerkinElmer Life Sciences. All other experimental reagents were from Sigma.

##### Plasmids and Mutagenesis

All plasmids used encoded either human, mouse, or rat FFA1–4 receptors with enhanced yellow fluorescent protein (eYFP) fused to their C termini in the pcDNA5 FRT/TO expression vector as described previously (11, 13, 17, 18). A chimeric form of hFFA2, in which extracellular loop 2 (ECL2) was replaced with ECL2 from hFFA3 was also as reported previously (13). All individual point mutations described were introduced using the QuikChange method (Stratagene).

##### Cell Culture, Transfection, and Stable Cell Lines

For experiments utilizing transient heterologous expression, HEK293T cells were used. Cells were maintained in Dulbecco's modified Eagle's medium (DMEM) supplemented with 10% FBS at 37 °C and 5% CO_2_. Transfections were carried out using polyethyleneimine, and experiments were conducted 48 h post-transfection. In experiments where stable cell lines were employed, the Flp-In^TM^ T-REx^TM^ system (Invitrogen) was used to generate 293 cells with tetracycline- or doxycycline-inducible expression of the receptor of interest. To generate these cell lines, Flp-In^TM^ T-REx^TM^ 293 cells were co-transfected with the pOG44 vector and the receptor of interest in pcDNA5/FRT/TO. Transfection with pOG44 drives expression of Flp recombinase, which, in turn, allows for recombination between FRT sites in pcDNA5/FRT/TO and in the genome of the Flp-In^TM^ T-REx^TM^ 293 cells, thus allowing identification of stable inducible cells for the receptor of interest to be generated by appropriate antibiotic selection. All experiments carried out using these cells were conducted after a 24-h treatment with 100 ng/ml doxycycline to induce expression of the receptor of interest.

For experiments using differentiated mouse adipocytes, subconfluent 3T3-L1 fibroblasts were maintained in DMEM supplemented with 10% newborn calf serum and incubated at 37 °C and 10% CO_2_. At 2 days postconfluence, growth-arrested cells were differentiated to 3T3-L1 adipocytes by the addition of DMEM containing 10% FBS, 0.5 mm methyl isobutylxanthine, 0.25 μm dexamethasone, 1 μg/ml bovine insulin, and 5 μm troglitazone. After a 72-h incubation (day 3), cells were supplemented with DMEM containing 10% FBS, 1 μg/ml bovine insulin, and 5 μm troglitazone. At day 6, the cells were changed into DMEM containing 10% FBS for 2 days, prior to use 9–14 days postdifferentiation.

The human-derived liposarcoma cell line SW872 was maintained in DMEM supplemented with 10% FBS and maintained at 37 °C and 10% CO_2_. To differentiate these cells into adipocytes, cells were plated in 12-well plates and cultured until confluent. Culture medium was then replaced with medium supplemented with 0.5 mm methyl isobutylxanthine, 1 μm dexamethasone, 10 μg/ml bovine insulin, and 500 μm oleic acid. After a 72-h incubation, culture medium was replaced with medium containing 10 μg/ml bovine insulin and 500 μm oleic acid. Culture medium was then replaced every 48 h with fresh medium supplemented only with 500 μm oleic acid until cells were used for experiments between days 10 and 14. To confirm differentiation, cells were fixed with 4% paraformaldehyde and stained with oil red O to demonstrate lipid accumulation.

STC-1 enteroendocrine cells were maintained in DMEM supplemented with 10% FBS and maintained at 37 °C and 10% CO_2_. For experiments, cells were plated in 24-well plates and cultured for at least 24 h before initiating GLP-1 secretion experiments.

##### [^35^S]GTPγS Incorporation Assay

Cell membrane preparations were generated from Flp-In^TM^ T-REx^TM^ 293 cells induced to express the receptor of interest, as described previously (18). [^35^S]GTPγS binding experiments were performed according to a method described previously (10). Briefly, cell membrane preparations containing 5 or 10 μg of protein were added to assay buffer (50 mm Tris-HCl, pH 7.4, 10 mm MgCl_2_, 100 mm NaCl, 1 mm EDTA, 1 μm GDP, and 0.1% fatty acid-free bovine serum albumin) containing the appropriate concentrations of ligand and allowed to reach equilibrium by preincubating for 15 min at 25 °C. To initiate the assay, 50 nCi of [^35^S]GTPγS was added to each tube, and the reaction was terminated by rapid filtration through GF/C glass filters using a 24-well Brandel cell harvester (Alpha Biotech, Glasgow, UK) after a 1-h incubation at 25 °C. Unbound [^35^S]GTPγS was washed from filters by three washes with ice-cold wash buffer (50 mm Tris-HCl, pH 7.4, and 10 mm MgCl_2_) before the remaining bound [^35^S]GTPγS was measured by liquid scintillation spectrometry.

##### Bioluminescence Resonance Energy Transfer (BRET) β-Arrestin-2 Recruitment Assay

A plasmid encoding an eYFP-tagged form of the receptor to be assayed was co-transfected in a 4:1 ratio with a β-arrestin-2 *Renilla* luciferase (Rluc) plasmid. Cells were then transferred into white 96-well plates 24 h post-transfection. Then 48 h post-transfection, cells were washed, and the culture medium was replaced with Hanks' balanced salt solution (HBSS) immediately prior to conducting the assay. To measure β-arrestin-2 recruitment, the Rluc substrate coelenterazine h was added to a final concentration of 2.5 μm, and then cells were incubated for 10 min at 37 °C, test compounds were added, and cells were incubated for a further 5 min at 37 °C. BRET resulting from receptor-β-arrestin-2 interaction was then assessed by measuring the ratio of luminescence at 535 and 475 nm using a Pherastar FS fitted with the BRET1 optic module (BMG Labtech, Aylesbury, UK).

##### Intracellular Ca^2+^ Mobilization Assay

All Ca^2+^ experiments were carried out using Flp-In^TM^ T-REx^TM^ stable inducible cell lines treated with doxycycline (100 ng/ml) to induce expression of the receptor of interest. Cells were plated 50,000/well in black 96-well plates with clear bottoms and then allowed to adhere for 3–6 h. Doxycycline was then added to induce receptor expression, and cells were maintained in culture overnight. Prior to the assay, cells were labeled for 45 min with the calcium-sensitive dye Fura-2 AM and then washed and incubated for 15 min with HBSS. Fura-2 fluorescent emission at 510 nm resulting from 340- or 380-nm excitation was then monitored using a Flexstation (Molecular Devices, Sunnyvale, CA) plate reader. Base-line fluorescence was measured for 16 s, test compounds were then added, and fluorescence was measured for an additional 74 s. The base line-subtracted maximum 340/380-nm ratio obtained after the compound addition was then used to plot concentration-response data.

##### Extracellular Signal-regulated Kinase 1/2 (ERK) Assay

All ERK phosphorylation experiments were carried out using Flp-In^TM^ T-REx^TM^ stable inducible cell lines treated with doxycycline (100 ng/ml) to induce expression of the receptor of interest. Briefly, 75,000 cells were seeded per well in a 96-well plate and then allowed to attach for 3–6 h before the addition of doxycycline to induce receptor expression. After incubating overnight, the culture medium was replaced with serum-free DMEM containing doxycycline (100 ng/ml), and cells were then incubated for a further 5–6 h prior to the assay. To conduct the assay, test compounds were added, and the cells were incubated at 37 °C for 5 min before the cells were lysed and assayed for phospho-ERK using an Alphascreen-based detection kit according to the manufacturer's protocol (PerkinElmer Life Sciences).

##### Reverse Transcription-PCR

To assess FFA2 and FFA3 transcript expression in SW872, 3T3-L1, and STC-1 cell lines, reverse transcription-PCR was carried out. Total RNA was first isolated from cells using an RNeasy isolation kit (Qiagen). Total RNA was treated with DNase I to eliminate any DNA contamination and then used in reverse transcription reactions with random decamer primers in order to generate cDNA. The cDNA was used in standard PCRs with primers for mFFA2 (forward, CGAGAACTTCACCCAAGAGC; reverse, TGAGGGAACTGAACACCACA); mFFA3 (forward, CCCAGTGGCTGTGGACTTAC; reverse, CAGAAAACGTTCGATGCTCA); hFFA2 (forward, TCTGCTACTGGCGTTTTGTG; reverse, AGGTGGGACACGTTGTAAGG); or hFFA3 (forward, GCAGCGTGGTCTACGTCATA; reverse, CGACATGGGACACGTTGTAG). Because FFA2 and FFA3 are both intronless, negative controls were carried out, where the reverse transcriptase enzyme was omitted (−RT), and positive controls were included using either hFFA2 or hFFA3 plasmid DNA or commercially obtained mouse genomic DNA.

##### SW872 and 3T3-L1 Lipolysis Assays

Differentiated SW872 or 3T3-L1 adipocytes were washed three times with HBSS prior to the co-addition of forskolin (10 μm; SW872) or isoprenaline (10 nm; 3T3-L1) to promote lipolysis, along with the test compound to be assessed. Cells were incubated at 37 °C for 2 h (SW872) or 1 h (3T3-L1), and then cell supernatants were transferred to microcentrifuge tubes. Glycerol concentration in supernatants was measured by dispensing 50 μl/well in triplicate into 96-well plates, followed by the addition of 50 μl/well free glycerol reagent (Sigma). Plates were then incubated at room temperature protected from light for 15 min before absorbance at 540 nm was measured using a Pherastar FS microplate reader (BMG Labtech).

##### GLP-1 Secretion

STC-1 cells were washed with HBSS supplemented with 20 mm HEPES before the addition of test compound in HBSS/HEPES containing the DPPIV inhibitor KR-62436 (2.5 μm) to prevent peptidase activity and the hydrolysis of GLP-1. Cells were incubated at 37 °C for 1 h before cell supernatants were collected in microcentrifuge tubes. Supernatants were then centrifuged to eliminate any cellular debris and assayed for GLP-1 concentration using an active GLP-1 ELISA kit (Millipore).

##### Molecular Modeling

The homology model of hFFA2 was taken from our previous study (10). Docking of compounds 1 and 2 into the model was performed using Glide 5.7 (Schrödinger, LLC, New York) of the Schrödinger computer package. The Glide docking box was defined in the cavity between transmembrane helices 3, 5, and 6, involving residues at positions 5.39, 7.35, and 6.55 (in the Ballesteros and Weinstein numbering system (19)), because we have shown that these residues are critical to anchor the carboxyl group of fatty acids (18). The Glide default settings with the extra-precision scoring option were used for docking. Images were prepared using the Maestro 9.2 interface.

##### Data Analysis and Curve Fitting

All data presented represent means ± S.E. of at least three independent experiments. Data analysis and curve fitting were carried out using the GraphPad Prism software package version 5.0b. Concentration-response data were plotted on a log axis, where the untreated vehicle control condition was plotted at one log unit lower than the lowest test concentration of ligand and then fitted to three-parameter sigmoidal concentration-response curves. Statistical analysis of curve fit parameters was carried out by independently fitting the data from triplicate experiments and comparing the resulting curve fit values by *t* test or one-way analysis of variance as appropriate. Antagonism experiments carried out with multiple fixed concentrations of antagonist were fit where appropriate to a global Gaddum/Schild EC_50_ shift equation in order to estimate pA_2_ values for the antagonist.

## RESULTS

### 

#### 

##### Compounds 1 and 2 Are Potent and Selective FFA2 Agonists

We synthesized two compounds based on a 4-oxobutanoic acid backbone (compounds **1** and **2**; Fig. 1*A*) that are related to structures described in the patent literature as agonists of FFA2 (16) and examined their potency and selectivity to activate FFA2 across various functional assays. First, a [^35^S]GTPγS incorporation assay was employed to assess G_i/o_-mediated signaling (Fig. 1*B*). In this assay, compounds **1** and **2** both effectively stimulated incorporation of [^35^S]GTPγS into membranes of Flp-In^TM^ T-REx^TM^ 293 cells induced to express hFFA2-eYFP with pEC_50_ values of 7.14 ± 0.08 and 6.98 ± 0.12, respectively. These compounds were significantly (*p* < 0.001) more potent than the endogenous ligand, **C3** (pEC_50_ = 4.27 ± 0.10), but had efficacy similar to that of **C3** (114 ± 4 and 94 ± 4% of the **C3** response, respectively). Because the closely related receptor FFA3 is also a G_i_-coupled GPCR, the potential of **1** and **2** to stimulate [^35^S]GTPγS incorporation into equivalent membranes from Flp-In^TM^ T-REx^TM^ 293 cells induced to express hFFA3-eYFP was used to determine their selectivity for hFFA2 over hFFA3 (Fig. 1*C*). In these experiments, although **C3** promoted binding of [^35^S]GTPγS with the anticipated potency (pEC_50_ = 3.62 ± 0.07), neither compound **1** nor **2** produced a measurable response at up to 10 μm, indicating that both compounds are highly selective for hFFA2 over hFFA3.

To examine the ability of **1** and **2** to stimulate G_q/11_-mediated pathways via activation of hFFA2, changes in intracellular Ca^2+^ were measured in intact Flp-In^TM^ T-REx^TM^ 293 cells induced to express hFFA2-eYFP (Fig. 1*D*). As with the [^35^S]GTPγS assay, both **1** and **2** did so and with significantly higher potency (pEC_50_ = 6.68 ± 0.06 and 6.39 ± 0.06, respectively) than **C3** (pEC_50_ = 3.50 ± 0.10). However, in this case, both **1** and **2** were found to have significantly greater efficacy than **C3** (*p* < 0.01), with *E*_max_ values of 132 ± 4 and 130 ± 4% of the **C3** response, respectively. To assess further the selectivity of **1** and **2** for FFA2, Ca^2+^ assays were also employed in Flp-In^TM^ T-REx^TM^ 293 cells induced to express equivalent C-terminally eYFP-tagged forms of either of the long chain free fatty acid-sensitive GPCRs, hFFA1 and hFFA4, because both of these couple predominantly to G_q/11_ G proteins. Compared with the previously described FFA1-selective ligand TUG-424 (20), which elevated Ca^2+^ with pEC_50_ = 7.19 ± 0.06 in hFFA1-eYFP-expressing cells (Fig. 1*E*), **2** produced no measurable response at concentrations up to 10 μm, whereas **1** did generate a Ca^2+^ response but only at the highest concentrations tested (3–10 μm). Because FFA1 agonists have at times been shown to possess PPARγ activity, we also tested whether **1** displayed agonism or antagonism of PPARγ (testing performed by Cerep, Poitiers, France). In these experiments, **1** (10 μm) was found to have no measurable activity either as an agonist or antagonist (data not shown). When experiments were conducted on cells induced to express hFFA4-eYFP (Fig. 1*F*), the previously described FFA4-selective agonist TUG-891 (17) effectively increased intracellular Ca^2+^ (pEC_50_ = 6.68 ± 0.08), whereas neither **1** nor **2** had any effect at concentrations up to 10 μm, indicating that **1** and **2** are extremely selective for FFA2 over both FFA1 and FFA4.

FFA2 activation has also been linked to increased phosphorylation of ERK (13), a pathway that may be initiated via activation of G_i_ or G_q/11_, as well as by G protein-independent signaling pathways. Therefore, we explored ERK phosphorylation stimulated by **C3**, **1**, and **2** in cells induced to express hFFA2-eYFP (Fig. 1*G*). In these experiments, **C3** stimulated increased ERK phosphorylation (pEC_50_ = 3.56 ± 0.11), as did both **1** (pEC_50_ = 6.94 ± 0.16) and **2** (pEC_50_ = 6.48 ± 0.14). Both **1** and **2** were significantly more potent than **C3** (*p* < 0.001), but, as in the [^35^S]GTPγS assay, neither **1** or **2** displayed efficacy significantly different from that of **C3**. The final FFA2 signaling pathway examined was the recruitment of β-arrestin-2. Previous work has shown that activation of hFFA2 does stimulate recruitment of β-arrestin-2 (21), an event that may be linked with GPCR internalization and/or G protein-independent signaling. In order to assess this, a BRET-based assay was employed in which hFFA2-eYFP and β-arrestin-2 *Renilla* luciferase were co-expressed transiently in HEK293T cells (Fig. 1*H*). In this assay, **C3** stimulated β-arrestin-2 recruitment to human FFA2-eYFP (pEC_50_ = 3.22 ± 0.10), and so did both **1** (pEC_50_ = 5.72 ± 0.10) and **2** (pEC_50_ = 5.35 ± 0.09). As in the other assays, both **1** and **2** were significantly more potent than **C3** (*p* < 0.001), and, although not statistically significant (*p* > 0.05), there was a trend toward increased efficacy for both **1** and **2** compared with **C3** in this assay.

##### Compounds 1 and 2 Bind to the Orthosteric Pocket of FFA2

**FIGURE 2.**
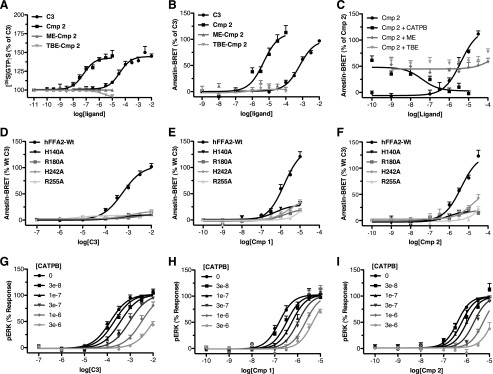
**Compounds 1 and 2 are orthosteric agonists of FFA2.** Concentration-response curves at hFFA2 for **C3**, **2,** a methyl ester of **2** (*ME-Cmp 2*), and a *tert*-butyl ester of **2** (*TBE-Cmp 2*) are shown in [^35^S]GTPγS (*A*) and β-arrestin-2 recruitment (*B*) assays. The ability of the hFFA2 antagonist CATPB as well as methyl and *tert*-butyl esters of **2** to inhibit an EC_50_ concentration of compound **2** in the β-arrestin-2 recruitment assay is shown in *C*. Concentration-response curves were generated using wild type and H140A, R180A, H242A, and R255A mutants of hFFA2 in the β-arrestin-2 recruitment assay, and the results are shown for **C3** (*D*), **1** (*E*), and **2** (*F*). Antagonism experiments where concentration-response curves were generated to agonist in the presence of increasing fixed concentrations of CATPB are shown using **C3** (*G*), **1** (*H*), and **2** (*I*) as agonists. *Error bars*, S.E.

The only series of synthetic small molecule agonists previously published and with reasonable potency at FFA2 are allosteric in nature and, as such, bind to a different site than the SCFAs. These compounds, exemplified by 4-CMTB, display reduced signaling in some pathways and lack the carboxylate moiety that is critical in the SCFAs for interaction with the orthosteric binding site (13). Because **1** and **2** both contain a carboxylate functionality and display broadly similar signaling responses to **C3** across all assays tested, we hypothesized that these compounds are probably orthosteric agonists of FFA2. To assess this, we first examined whether the carboxylate was critical to ligand function by generating both methyl and *tert*-butyl ester analogs of **2**. Both of these compounds lacked activity at hFFA2 in either [^35^S]GTPγS incorporation (Fig. 2*A*) or β-arrestin-2 interaction (Fig. 2*B*) assays, indicating the critical importance of the carboxylate to ligand function. To confirm that this reduced function was due to reduced binding of the ester ligands, we performed potential antagonism experiments with each of the esters against an EC_50_ concentration of **2** (Fig. 2*C*). In such experiments, although the response to **2** was antagonized by the previously reported hFFA2 antagonist/inverse agonist CATPB (11), no blockade of the effect was observed with either ester up to 100 μm, indicating that the ester modifications resulted in reduced binding affinity for these ligands rather than converting agonism into antagonism. Because recognition of the carboxylate moiety of the SCFAs requires arginine and histidine residues located in each of transmembrane helices IV–VII of FFA2 (18), we next assessed the activity of compounds **1** and **2** at hFFA2 point mutants of each of these residues: H140A^4.56^ (Ballesteros and Weinstein nomenclature in superscript), R180A^5.39^, H242A^6.55^, and R255A^7.35^. As anticipated, when using β-arrestin-2 interaction assays, activity of **C3** was virtually abolished at each of these modified receptors (Fig. 2*D*), and the activity of both **1** (Fig. 2*E*) and **2** (Fig. 2*F*) was also greatly reduced in efficacy and/or potency, indicating that interaction with these residues is important for function of these ligands. As additional support for an orthosteric mode of binding of **1** and **2**, we compared the ability of CATPB to limit the function of **C3**, **1**, and **2**. In ERK phosphorylation assays, increasing concentrations of CATPB resulted in higher concentrations of each agonist being required to cause half-maximal promotion of phosphorylation (Fig. 2, *G*, *H*, and *I*). Furthermore, in each case, the effect of CATPB was fully overcome by sufficiently high concentrations of the agonist. Global curve fitting of the data yielded similar pA_2_ values for CATPB of 7.23 ± 0.09, 7.58 ± 0.15, and 7.24 ± 0.14 for experiments conducted with **C3**, **1**, and **2**, respectively. These data are consistent with competitive interactions between each agonist and CATPB and, therefore, with **C3**, **1**, and **2** sharing an overlapping binding site.

##### Compounds 1 and 2 Are Not Modulated Allosterically by 4-CMTB

**FIGURE 3.**
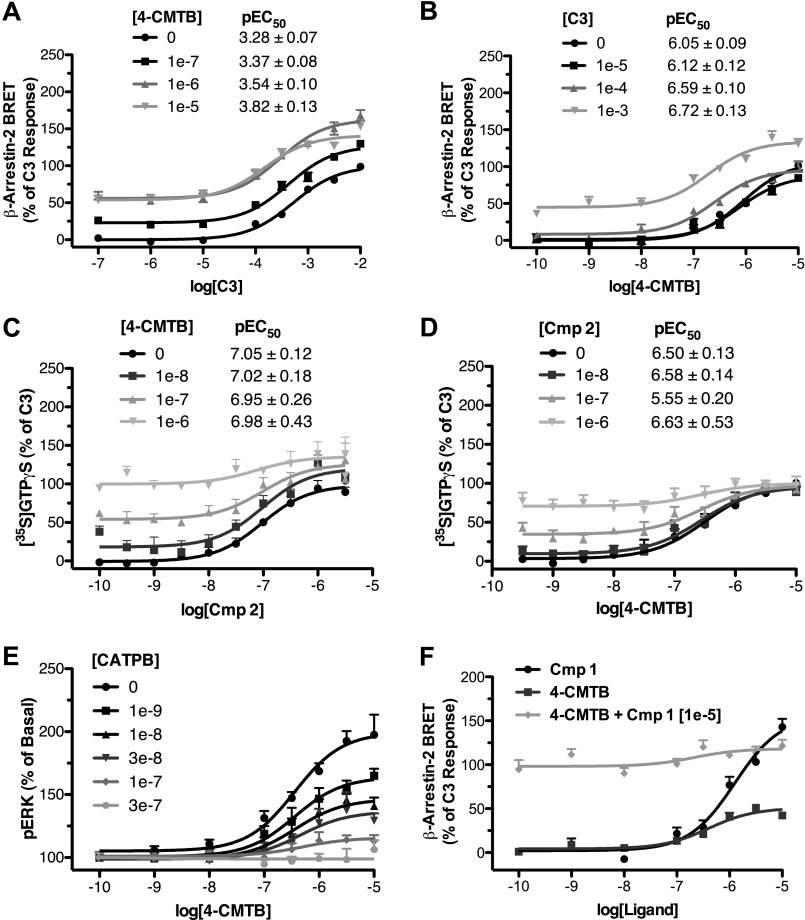
**The FFA2 allosteric ligand 4-CMTB does not modulate function of compounds 1 and 2.** Concentration-response curves for **C3** in the β-arrestin-2 recruitment assay in the presence of increasing fixed concentrations of 4-CMTB are shown in *A*. The reciprocal experiment showing the concentration response to 4-CMTB with increasing fixed concentrations of **C3** is shown in *B*. Similar experiments are shown using a [^35^S]GTPγS assay with **2** and 4-CMTB (*C* and *D*). An antagonism experiment measuring the concentration response to 4-CMTB in a pERK assay in the presence of increasing fixed concentrations of CATPB is shown in *E*. In *F*, a competition experiment is shown using a fixed concentration (10 μm) of the full agonist **1** with increasing concentrations of the partial agonist 4-CMTB in the β-arrestin-2 BRET assay. *Error bars*, S.E.

There were, however, differences between the behavior of compounds **1** and **2** and **C3**. The compound 4-CMTB is recognized as an ago-allosteric modulator of FFA2 (12, 13). As a result, as well as direct activation of signaling, increasing submaximal, fixed concentrations of 4-CMTB increase the potency of **C3** (Fig. 3*A*), and *vice versa* (Fig. 3*B*). By contrast, increasing fixed concentrations of 4-CMTB did not alter the potency of **2** (Fig. 3*C*), nor did fixed concentrations of **2** alter the potency of 4-CMTB (Fig. 3*D*). This observation could be consistent with either of two possibilities: 1) with so-called “probe dependence” (22) of 4-CMTB allosterism, such that it modulates the binding of **C3** but not **2**, or 2) with the binding sites of 4-CMTB and **2** overlapping each other. Although **C3** and **2** appear to bind to the same site, whereas 4-CMTB and **C3** do not, given the very small size of **C3** relative to **2**, it is still at least conceivable that **2** could overlap the binding sites of both **C3** and 4-CMTB. To address this possibility, we first examined whether the hFFA2 antagonist CATPB was competitive with 4-CMTB by measuring the 4-CMTB concentration response in the presence of increasing concentrations of CATPB (Fig. 3*E*). Interestingly, in these experiments, CATPB did inhibit the 4-CMTB response; however, unlike with **C3**, **1**, and **2**, where the inhibition resulted in reduced potency with no effect on efficacy, the inhibition of 4-CMTB by CATPB had the opposite effect, reducing efficacy with no effect on potency. Because we had previously shown that CATPB is competitive with **C3**, **1**, and **2** and therefore does not bind irreversibly, this pattern of CATPB inhibition of the ERK response to 4-CMTB indicates that these two molecules must bind to separate sites and that CATPB is a negative allosteric modulator of 4-CMTB efficacy, further strengthening the case that **2** and 4-CMTB bind to distinct sites. Finally, to examine this more directly, we took advantage of the fact that 4-CMTB is only a partial agonist in the BRET assay. Therefore, if competitive, 4-CMTB would be expected to antagonize the response to the full agonist, **1**, measured in this assay (Fig. 3*F*). In these experiments, **1** produced a maximal response that was 156 ± 12% of the **C3** response, whereas 4-CMTB was a partial agonist producing a maximal response that was only 51 ± 4% of the **C3** maximum. However, when increasing concentrations of 4-CMTB were used with a fixed, high concentration of **1**, there was no inhibition, and indeed there was a small increase in response, with increasing concentrations of 4-CMTB. Taken together, these results suggest strongly that both **1** and **2** are binding to a distinct site to 4-CMTB and that, therefore, the lack of allosterism between these two compounds represents a probe dependence in the ability of 4-CMTB to modulate function of FFA2 orthosteric agonists.

##### ECL2 Is Critical to the Binding and Selectivity of Compounds 1 and 2

**FIGURE 4.**
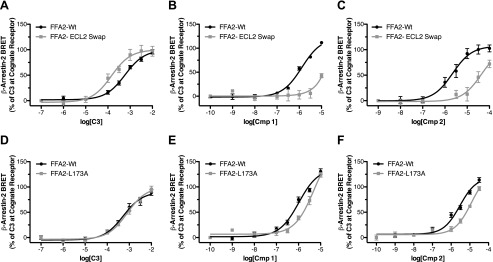
**Extracellular loop 2 is critical to the function of compounds 1 and 2 at hFFA2.** Concentration responses to **C3** (*A*), **1** (*B*), and **2** (*C*) are shown using the β-arrestin-2 recruitment assay for wild type hFFA2 as well as for a chimeric form of hFFA2 where its ECL2 has been replaced with the ECL2 of hFFA3. Similar experiments show concentration responses to **C3**, **1**, and **2** (*D–F*) at wild type hFFA2 and the L173A point mutant of hFFA2. *Error bars*, S.E.

We next considered whether the probe-dependent effect of 4-CMTB might be instructive in defining the mode or location of binding of **1** and **2**. A recent study demonstrated that replacement of ECL2 of hFFA2 with the equivalent region from hFFA3 has no negative effect on the potency of **C3** or 4-CMTB but essentially eliminates allosteric communication between the binding sites of these ligands (13). We considered, therefore, if the lack of allosteric interactions between 4-CMTB and **2** reflects that these ligands interact with ECL2 in such a way as to prevent allosteric communication. We tested this by comparing the potency of **C3**, **1**, and **2** in a β-arrestin-2 recruitment assay at wild type hFFA2 and the variant in which ECL2 is replaced by this sequence from hFFA3 (13). As observed previously, this modification resulted in a modest increase in the potency of **C3**, pEC_50_ of 3.84 ± 0.013 at the ECL2 swap mutant compared with 3.23 ± 0.07 at wild type (Fig. 4*A*). By contrast, for both **1** (Fig. 4*B*) and **2** (Fig. 4*C*), marked reductions in potency were observed. In addition to showing broadly that ECL2 is important for allosteric communication between **C3** and 4-CMTB, Smith *et al.* (13) also identified a single point mutation in ECL2 of hFFA2, L173A, which also eliminated allosteric communication. This alteration did not affect the potency of **C3** in the β-arrestin-2 interaction assay (Fig. 4*D*) but resulted, however, in a significant (*p* < 0.01) decrease in potency for both **1** (pEC_50_ of 5.29 ± 0.11 at L173A compared with 5.99 ± 0.07 at wild type (Fig. 4*E*)) and **2** (pEC_50_ values of 4.93 ± 0.11 and 5.54 ± 0.08 at L173A and wild type, respectively (Fig. 4*F*)).

**FIGURE 5.**
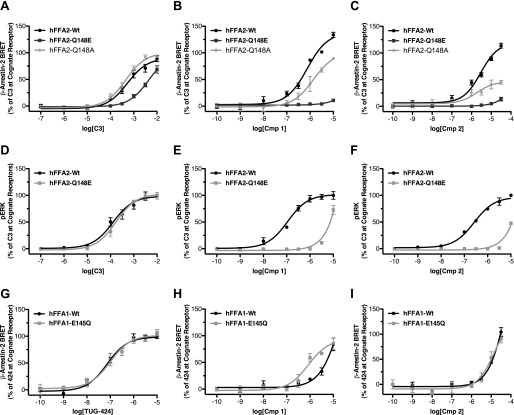
**The absence of a glutamate residue in ECL2 of FFA2 that is present in both FFA1 and FFA3 defines the FFA2 selectivity of compound 1 and 2.** Concentration responses are shown using the β-arrestin-2 recruitment assay for **C3** (*A*), **1** (*B*), and **2** (*C*) at wild type, Q148E, and Q148A variants of hFFA2. Similar pERK experiments are shown with wild type and Q148E hFFA2 (*D–F*). β-Arrestin-2 recruitment to wild type and the E145Q mutant of hFFA1 is shown for TUG-424 (*G*), **1** (*H*), and **2** (*I*). *Error bars*, S.E.

Because the hFFA2/hFFA3 ECL2 swap mutant substantially lost potency to both **1** and **2**, we next explored if ECL2 represents a key region in defining the selectivity of these compounds for hFFA2. To do so, we employed sequence alignment to identify residues in ECL2 conserved between FFA1 and FFA3, but not FFA2. This search yielded one obvious residue, where a glutamic acid is present in hFFA1 (position 145) and hFFA3 (position 152) but is a glutamine in hFFA2 (position 148). Previous work has shown that alteration of Glu-145 to alanine in FFA1 breaks an extracellular ionic lock and increases ligand-independent constitutive activity (23). We therefore generated both Q148E and Q148A mutants of hFFA2 to test whether a similar ionic lock might influence the FFA2 selectivity of **1** and **2**. These mutants were first assessed in the β-arrestin-2 assay with **C3** (Fig. 5*A*), **1** (Fig. 5*B*), and **2** (Fig. 5*C*). Although the potency of **C3** was reduced some 10-fold at the Q148E mutant (pEC_50_ = 3.27 ± 0.10 at wild type and 2.34 ± 0.11 at Q148E), both **1** and **2** were virtually inactive at this mutant. In contrast, **C3** did not show reduced potency at the Q148A mutant (pEC_50_ = 3.35 ± 0.08), and although both had reduced efficacy, neither **1** nor **2** displayed significant loss in potency at the Q148A mutant. Because the potency of agonists in the β-arrestin-2 assay generally reflects receptor occupancy and hence is a surrogate measure of affinity, this suggests that the presence of a glutamate but not alanine at this position significantly reduces the binding of both **1** and **2** to hFFA2. To explore this further, we also employed the ERK assay because this often provides higher agonist potency values due to the effects of receptor reserve (24). Here, the potency of **C3** (Fig. 5*D*) was little affected at Q148E compared with wild type hFFA2 (pEC_50_ = 3.89 ± 0.11 for wild type and 3.76 ± 0.07 at Q148E), whereas for both **1** (Fig. 5*E*) and **2** (Fig. 5*F*), there was again a nearly complete loss of agonist function. These results confirm that the lack of glutamate at this position in FFA2 is critical to the binding of **1** and **2** and therefore potentially could be involved in the selectivity of these compounds. To test whether this position contributes to the selectivity, we generated the reciprocal E145Q and E154Q mutants of hFFA1 and hFFA3, respectively. Interestingly, although the E145Q mutant did not alter the potency of the FFA1 agonist TUG-424 in a β-arrestin-2 recruitment assay (Fig. 5*G*), it did result in a clear gain of function for **1** when compared against wild type hFFA1 (Fig. 5*H*) but did not significantly alter the response for **2** (Fig. 5*I*). Similar experiments were conducted on the equivalent E154Q mutant of hFFA3 in [^35^S]GTPγS experiments; however, this mutant was found to completely lack function to all ligands tested (data not shown).

##### Molecular Modeling Suggests Further Interactions within the Transmembrane Domains of hFFA2 for Compounds 1 and 2

**FIGURE 6.**
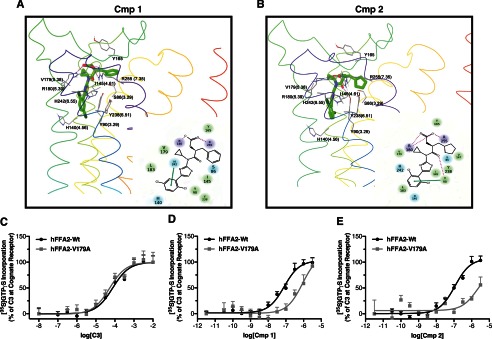
**Molecular modeling and mutagenesis reveals key transmembrane residues involved in the binding of compounds 1 and 2 (*Cmp 1* and *Cmp 2*).** *A* and *B*, the predicted binding pose of compounds **1** and **2** in the hFFA2 homology model and two-dimensional schematic representations of these are displayed. Residues forming contacts with the ligands are shown as *gray sticks*, and the receptor Cα atoms are *colored* based on the residue position within individual transmembrane domains. In the scheme to the *right* of each homology model, the residues are *colored* based on their physicochemical properties and *scaled* based on the distance to the two-dimensional plane. Hydrogen bonds and π-π aromatic stacking are indicated by *dotted pink* and *continuous green lines*, respectively. *Error bars*, S.E.

Predictions and modeling of extracellular loop regions of GPCRs remain highly speculative and challenging, particularly for receptors well removed phylogenetically from those for which atomic level structures are available. By contrast, the overall architecture of the transmembrane helix bundle is generally well preserved among GPCRs. As such, confidence in homology models of these regions is increasing, and well validated models are becoming widely used in suggesting likely ligand-receptor interactions within these regions. FFA2 homology models have provided important insights into modes of orthosteric ligand binding and differences in this between species orthologs (13, 18, 21). We therefore employed homology modeling of hFFA2 and ligand docking methods to suggest additional residues that might contribute to the enhanced potency of **1** and **2** compared with **C3** (Fig. 6, *A* and *B*). Based on such models, five transmembrane residues and one additional ECL2 residue were highlighted for subsequent analysis. Each of these was mutated to alanine and assessed with **C3**, **1**, and **2** in a [^35^S]GTPγS incorporation assay (Table 1). From these studies, it was apparent that several mutants (*i.e.* Y90A^3.29^, Y165A^ECL2^, and Y238A^6.51^) displayed significantly lower potency to each ligand, indicating their likely contribution to the orthosteric binding pocket by forming hydrophobic and aromatic interactions. In addition, Tyr-165 might also contribute in steering the ligand into the binding cavity. The I145A^4.61^ mutant appeared to not be expressed and produced no response to any ligand. Most interestingly in the current context, although the V179A^5.38^ mutant did not alter potency for **C3** (Fig. 6*C*), it did result in a significant reduction in potency for both **1** (*p* < 0.01; Fig. 6*D*) and **2** (*p* < 0.05; Fig. 6*E*). Our docking results predict that the cyclopropyl ring of **1** and **2** forms hydrophobic contacts with V179A^5.38^. Overall, the ligands **1** and **2** have more contacts with the residues of helices 3, 5, and 6 in the extracellular part of the helical bundle that remarkably impact the potency of the ligands when the residues are mutated to alanine.

**TABLE 1:** **Potency of C3, 1, and 2 in a [^35^S]GTPγS assay to activate FFA2 and FFA2 mutants**

Mutant	Expression*^a^*	C3*^b^*	Compound 1*^b^*	Compound 2*^b^*
hFFA2	100	4.12 ± 0.12 (100)	7.10 ± 0.12 (101 ± 6)	6.94 ± 0.09 (107 ± 5)
Y90A	81 ± 9	2.35 ± 0.26 (43 ± 11)	<5.5	<5.5
I145A	2.6 ± 2	NR*^c^*	NR	NR
Y165A	33 ± 3	2.87 ± 0.47 (30 ± 12)	6.45 ± 0.42 (40 ± 13)	<5.5
V179A	100 ± 6	4.28 ± 0.16 (48 ± 3)	6.06 ± 0.25 (55 ± 11)	5.67 ± 0.57 (42 ± 24)
L183A	14 ± 1	4.04 ± 0.14 (72 ± 4)	7.16 ± 0.20 (83 ± 8)	6.79 ± 0.18 (81 ± 8)
Y238A	40 ± 2	2.76 ± 0.26 (38 ± 8)	<5.5	<5.5

*^a^* eYFP signal measured in membranes expressing each mutant expressed as the percentage of the eYFP fluorescence signal measured from the hFFA2 WT membranes.

*^b^* pEC_50_ values are reported with efficacy expressed as a percentage of the **C3** response at wild type hFFA2 in parenthesis.

*^c^* NR, no response.

##### Compounds 1 and 2 Display Differential Function at Rodent Orthologs of FFA2

**FIGURE 7.**
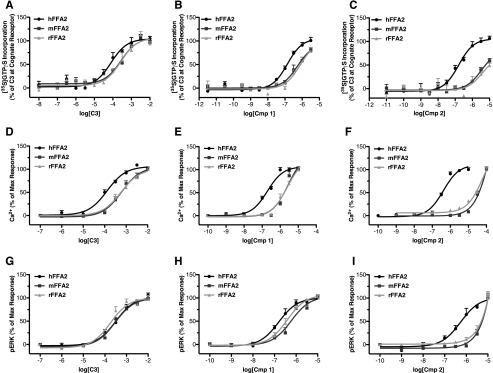
**Compound 2 and to a substantially lesser extent compound 1 shows reduced potency at rodent orthologs of FFA2.** Concentration responses for **C3** (*A*, *D*, and *G*), **1** (*B*, *E*, and *H*), and **2** (*C*, *F*, and *I*) are shown at human, mouse, and rat orthologs of FFA2 in [^35^S]GTPγS (*A–C*), Ca^2+^ (*D–F*), and pERK (*G–I*) assays. *Error bars*, S.E.

Recent work has demonstrated that FFA2 displays significant species ortholog variation with respect to both the endogenous SCFA ligands and to the synthetic antagonist CATPB (11, 21). Considering this, it was important to establish whether similar species ortholog variation might affect the usefulness of **1** and **2** in non-human systems. For this, we compared the activity of **C3**, **1**, and **2** at each of the human, mouse, and rat orthologs of FFA2 across multiple assay end points (Table 2). Initially, we examined the responses of **C3** (Fig. 7*A*), **1** (Fig. 7*B*), and **2** (Fig. 7*C*) at each ortholog in the G_i/o_-dependent [^35^S]GTPγS assay. Consistent with previous reports (11), **C3** displayed significantly (*p* < 0.01) lower potency at mFFA2 and rFFA2 compared with hFFA2. Interestingly, although **1** also displayed a reduction in potency (*p* < 0.05) of approximately the same magnitude as **C3** at mFFA2 (3.9-fold) and rFFA2 (5.4-fold) compared with hFFA2, **2** displayed a much larger reduction in potency at both mFFA2 (20-fold) and rFFA2 (22-fold) compared with hFFA2. Similar results were obtained when these compounds were assessed at the species orthologs in a Ca^2+^ assay to measure G_q/11_-coupled signaling (Fig. 7, *D–F*), with a much greater reduction in potency observed for **2** than for **C3** or **1**. In addition, because **1** was found to have some activity at hFFA1, we also tested these compounds at mFFA1 in a Ca^2+^ assay, finding no activity for **2** and only weak activity for **1**, requiring at least 10 μm before any Ca^2+^ response was observed (data not shown).

**TABLE 2:** **Potency of C3, 1, and 2 at human and rodent orthologs of FFA2 across various functional assays**

	hFFA2	mFFA2	mSelect.*^a^*	rFFA2	rSelect.*^b^*
**[^35^S]GTPγS**					
**C3**	4.10 ± 0.12	3.57 ± 0.10	0.53	3.57 ± 0.13	0.53
**1**	6.86 ± 0.07	6.27 ± 0.13	0.59	6.13 ± 0.14	0.73
**2**	6.79 ± 0.10	5.48 ± 0.16	1.31	5.43 ± 0.26	1.36

**Ca^2+^**					
**C3**	3.89 ± 0.07	3.21 ± 0.09	0.68	3.24 ± 0.07	0.65
**1**	6.71 ± 0.07	5.57 ± 0.10	1.14	5.61 ± 0.07	1.10
**2**	6.39 ± 0.06	3.77 ± 0.17	2.62	4.20 ± 0.09	2.19

**pERK**					
**C3**	3.50 ± 0.10	3.52 ± 0.09	−0.02	3.74 ± 0.10	−0.24
**1**	6.80 ± 0.11	6.23 ± 0.12	0.57	6.41 ± 0.09	0.39
**2**	6.26 ± 0.12	<4.5	>1.76	<4.5	>1.76

*^a^* mSelect., the preference for ligand at hFFA2 over mFFA2. Values represent the difference in pEC_50_ obtained between species orthologs in the same assay.

*^b^* rSelect., the preference for ligand at hFFA2 over rFFA2. Values represent the difference in pEC_50_ obtained between species orthologs in the same assay.

**FIGURE 8.**
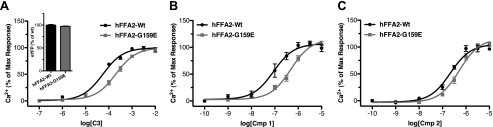
**The absence of an ionic lock in hFFA2 accounts for the greater potency of C3, 1, and to some extent 2 at the hFFA2 compared with the rodent orthologs.** Concentration responses for **C3** (*A*), **1** (*B*), and **2** (*C*) are shown in a Ca^2+^ mobilization assay using wild type and G159E hFFA2. The relative expression of wild type and G159E was assessed by measuring eYFP fluorescence and is shown as an *inset* to *A. Error bars*, S.E.

In ERK phosphorylation assays, similar potencies were observed for **C3** at hFFA2, mFFA2, and rFFA2 (Fig. 7*G*), and a small reduction in potency at the rodent orthologs was observed with **1** (Fig. 7*H*), whereas, once more, a substantial reduction in potency was seen for **2** (Fig. 7*I*). We have recently demonstrated that utilizing the variation in pharmacology between species orthologs of FFA2 can be a valuable approach in defining key aspects of receptor function (11, 21). Therefore, we next examined whether we could take advantage of the species differences in the function of **C3**, **1**, and **2** to help further define important and differential aspects of their function. First we considered a possible explanation for the broad decrease in potency we observed across all ligands. Recently, we have demonstrated that the absence in hFFA2, or presence in mFFA2, of an ionic lock between a glutamate residue at position 159 in ECL2 and arginine residues within the orthosteric binding pocket accounts for both reduced constitutive activity and lower SCFA potency at the mouse ortholog (11). Therefore, we hypothesized that this same residue might account for the broad decrease in potency we observed across all ligands. To assess this, we compared the ability of **C3** (Fig. 8*A*), **1** (Fig. 8*B*), and **2** (Fig. 8*C*) to produce a Ca^2+^ response via either wild type hFFA2 or a G159E hFFA2 mutant. In these experiments, despite similar levels of expression, **C3** displayed significantly reduced potency at the G159E mutant compared with wild type (pEC_50_ = 4.26 ± 0.06 at wild type and 3.69 ± 0.06 at G159E). Similarly, **1** displayed reduced potency (7.06 ± 0.11 at wild type compared with 6.32 ± 0.07 at G159E), as did **2** (6.66 ± 0.06 at wild type and 6.28 ± 0.07 at G159E). Interestingly, the magnitude of potency decreases observed at the G159E mutant were similar for **C3** (3.7-fold) and **1** (5.5-fold) to the potency decreases observed for these compounds at the two rodent orthologs compared with human FFA2 in the Ca^2+^ assay (∼4.5-fold for **C3** and ∼12-fold for **1**). In contrast, the potency decrease for **2** was only 2.4-fold at the G159E hFFA2 mutant, substantially less than the decrease observed for rodent compared with human orthologs in the Ca^2+^ assay using this ligand (416-fold for mFFA2 and 154-fold for rFFA2). These results indicate that the presence or absence of this “ionic lock” accounts for the broad decrease in potency seen for all agonist ligands at the rodent orthologs but does not account for the substantially greater loss in potency observed with **2**.

**FIGURE 9.**
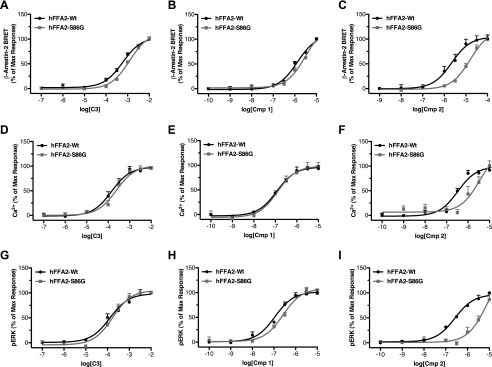
**The reduced function of compound 2 at rodent FFA2 orthologs results from the presence of glycine at position 86, compared with serine at this position in hFFA2.** Concentration-response curves are shown at wild type and S86G hFFA2 in β-arrestin-2 (*A–C*), Ca^2+^ mobilization (*D–F*), and pERK (*G–I*) assays using **C3**, **1**, or **2**. *Error bars*, S.E.

To attempt to understand this selective reduction in potency for **2**, we re-examined the homology models with **1** or **2** docked (Fig. 6, *A* and *B*) with the aim of identifying key residues predicted to be involved in ligand interaction in the human ortholog that were not conserved in the mouse and rat orthologs. This yielded only one residue in proximity to the defined orthosteric pocket, serine 86^3.29^ in hFFA2, which is glycine in both rFFA2 and mFFA2. We hence generated an S86G mutant of hFFA2 and examined its function across several assays. Initially, we tested this mutant in the β-arrestin-2 recruitment assay, where, although this alteration had no significant effect on the potency of either **C3** (Fig. 9*A*) or **1** (Fig. 9*B*), it did result in a significant reduction of potency of **2** (Fig. 9*C*). Similar results were observed when the S86G hFFA2 mutant was tested in both Ca^2+^ (Fig. 9, *D–F*) and ERK (Fig. 9, *G–I*) assays. Although the decreases in potency for **2** at S86G compared with wild type hFFA2 (7-fold in the β-arrestin-2 assay, 10-fold in the Ca^2+^ assay, and 8-fold in the pERK assay) were not as large as the decrease observed for **2** at the rodent ortholog compared with human FFA2, this site clearly contributes to the specific reduction in potency for **2** in the rodent orthologs. Indeed, it is likely that a combination of the effects of the presence of Gly-86 and Glu-159 residues in rat and mouse FFA2 accounts for the bulk of potency loss observed for **2** at the rodent orthologs.

##### Compounds 1 and 2 Demonstrate FFA2-mediated Inhibition of Lipolysis in Human Adipocytes, whereas Only Compound 1 Effectively Inhibits Lipolysis in Mouse Adipocytes

**FIGURE 10.**
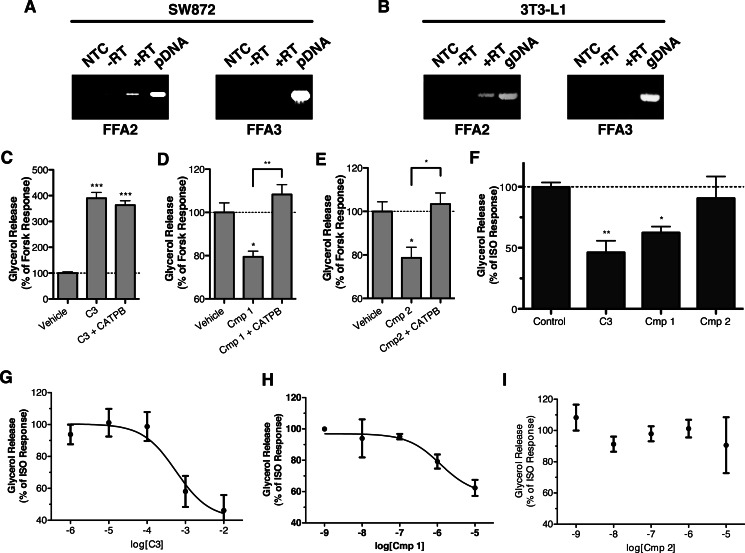
**Compounds 1 and 2 define the role of FFA2 in the inhibition of lipolysis in human- and murine-derived adipocyte cell lines.** Expression of FFA2 and FFA3 transcripts in differentiated SW872 and 3T3-L1 adipocytes was assessed by RT-PCR and is shown in *A* and *B*, respectively. No-template control reactions (*NTC*), reactions without reverse transcriptase (−*RT*), reactions with reverse transcriptase (+*RT*), and plasmid (*pDNA*)/genomic DNA (*gDNA*)-positive control reactions are shown. The effect of **C3** (10 mm), **1** (10 μm), and **2** (10 μm) on forskolin (*Forsk*) (10 μm)-stimulated lipolysis in SW872 is shown in *C–E*. In each case, the ability of the hFFA2 antagonist, CATPB (10 μm), to block ligand response is also shown. The effect of single, high concentrations of **C3** (10 mm), **1** (10 μm), and **2** (10 μm) to inhibit isoprenaline (*ISO*) (10 nm)-induced lipolysis in differentiated 3T3-L1 adipocytes is shown in *F*. **, *p* < 0.01; *, *p* < 0.05. Concentration responses are shown for **C3** (*G*), **1** (*H*), and **2** (*I*) in the 3T3-L1 inhibition of lipolysis assay. *Error bars*, S.E.

Having defined the potency and activity of **C3**, **1**, and **2** in cells transfected to express each of the human, mouse and rat orthologs of FFA2 in a heterologous manner, this provided the opportunity to define the specific role of FFA2 in functional responses of cells endogenously expressing the receptor. To do so, we examined the effects of these ligands on stimulated lipolysis in both a human-derived liposarcoma cell line, SW872, and in mouse 3T3-L1 cells, after differentiating each cell line into adipocyte-like phenotypes. Initial RT-PCR studies on mRNA isolated from differentiated SW872 and 3T3-L1 cells confirmed expression of FFA2 but not FFA3 in each cell type (Fig. 10, *A* and *B*).

To examine lipolysis in the differentiated SW872 line, cells were treated with 10 μm forskolin to stimulate lipolysis, which was then measured by assessing the concentration of glycerol released into the cell culture supernatant (Fig. 10, *C–E*). Unexpectedly, treatment with **C3** (10 mm) enhanced lipolysis in these cells markedly. However, this effect was not blocked by the hFFA2 antagonist CATPB. In contrast, both **1** (10 μm) and **2** (10 μm) significantly reduced (*p* < 0.05) glycerol release to 79 ± 9 and 79 ± 15% of the forskolin alone control, respectively. Importantly, for both **1** and **2**, the effect of the ligand was completely reversed by the hFFA2 antagonist CATPB.

To assess lipolysis in the 3T3-L1 adipocytes, we first treated cells with high concentrations of **C3** (10 mm), **1** (10 μm), or **2** (10 μm) and measured inhibition of isoprenaline-stimulated glycerol release (Fig. 10*F*). Both **C3** (*p* < 0.01) and **1** (*p* < 0.05) significantly inhibited glycerol release, whereas **2** did not. Although failing to reach statistical significance, **C3** tended to produce a greater inhibition of glycerol release, 54 ± 10%, compared with only 38 ± 0.5% for **1**. Finally, in concentration-response experiments, **C3** inhibited glycerol release with a pIC_50_ = 3.24 ± 0.36 (Fig. 10*G*), whereas **1** was some 480-fold more potent (pIC_50_ = 5.92 ± 0.43) (Fig. 10*H*), and **2** was again without significant effect (Fig. 10*I*). Because the only FFA2 antagonist currently available, CATPB, is highly human-selective, it was not possible to perform studies to block these effects with an FFA2 antagonist. However, because we had demonstrated that **1** has low potency agonism at FFA1, we did confirm that the effect of compound **1** was not blocked by the FFA1 antagonist GW1100 (data not shown). These results demonstrate that FFA2 inhibits lipolysis in both human- and rodent-derived adipocyte models and that, despite their chemical similarity, compound **1** but not compound **2** is a novel and valuable tool to specifically assess the function of FFA2 in murine cells and tissues.

##### Compound 1 Stimulates GLP-1 Release from Murine STC-1 Enteroendocrine Cells

**FIGURE 11.**
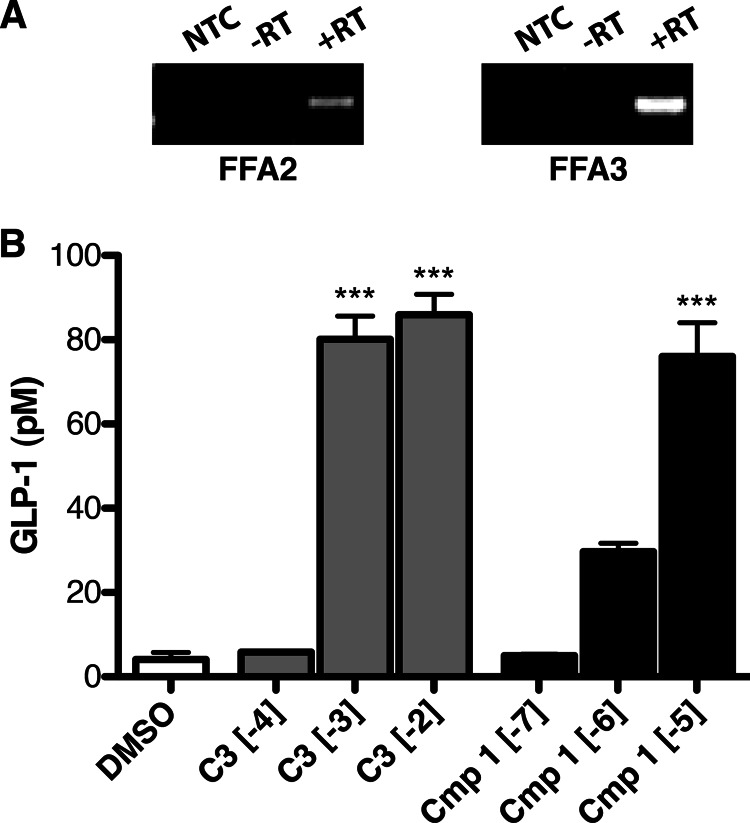
**Compound 1 stimulates GLP-1 release in the murine STC-1 enteroendocrine cell line.** Expression of FFA2 and FFA3 transcripts in STC-1 cells is shown in *A*. No-template control reactions (*NTC*), reactions without reverse transcriptase (−*RT*), and reactions with reverse transcriptase (+*RT*) are shown. The ability of varying log(*M*) concentrations **C3** (*gray bars*) or **1** (*black bars*) to stimulate GLP-1 secretion from these cells is shown in *B*. ***, *p* < 0.001 compared with DMSO vehicle treatment. *Error bars*, S.E.

Having shown that **1** can be used to assess FFA2 function in mouse cells endogenously expressing the receptor, we finally set out to use this compound to selectively examine FFA2 *versus* FFA3 function in cells endogenously co-expressing both receptors. For this, we examined the release of GLP-1 from the mouse-derived enteroendocrine cell line STC-1, which we confirmed by RT-PCR to express both FFA2 and FFA3 (Fig. 11*A*). Treatment of these cells with **C3** (1 or 10 mm) or **1** (10 μm) significantly increased GLP-1 secretion (*p* < 0.001). Interestingly, there was no difference in the maximum GLP-1 response produced by **C3** or **1**, indicating that **C3** is probably stimulating GLP-1 release primarily through FFA2 and not FFA3 in this cell line.

## DISCUSSION

In cases where closely related GPCRs respond to the same endogenous ligand(s), developing selective synthetic agonists or antagonists capable of differentiating between the receptors is critical to defining their individual biological roles. This is the case for FFA2 and FFA3 because each responds to the same endogenous SCFA ligands. Differences in the rank order of potency have been described for the SCFAs at FFA2 compared with FFA3 (4). However, the potency and selectivity of these compounds remains low. As a result, very high concentrations of SCFAs must be used, and, because the SCFAs are known to have additional off-target effects, including, for example, inhibition of histone deacetylases (25), this has made attributing the biological effects of SCFAs directly to FFA2 or FFA3 challenging. Further complicating the issue is the recent observation that C2, a SCFA with a degree of selectivity for hFFA2 over hFFA3, does not show this selectivity at the rodent orthologs of these receptors (11), making it effectively impossible to clearly define FFA2 *versus* FFA3 functions using SCFAs in rodent models without knock-out approaches. Although such knock-out studies have been informative in this respect (8, 9, 14, 26, 27), there is also evidence that knock-out of FFA3 results in reduced FFA2 expression (8), clearly complicating the interpretation of data based upon such animals. Moreover, in studies on the possible contribution of FFA2 to gut inflammatory disease, two distinct lines of FFA2 knock-out mice generated entirely contradictory results, in that in a model of colitis, one line displayed reduced and the other exaggerated inflammation (26, 27). To address such issues, there is a clear need for synthetic ligands that can effectively differentiate between FFA2 and FFA3 in both human and non-human experimental systems. Although several synthetic ligands have been reported with selectivity for either FFA2 or FFA3 (10–13, 28), each of these suffers from either poor potency, low selectivity, altered function due to an allosteric mode of action, or lack of function at non-human orthologs of the receptors. Therefore, our description of compounds **1** and **2** as the first potent and selective orthosteric agonists of hFFA2 and of **1** as the first orthosteric compound displaying reasonable activity at rodent orthologs of FFA2 suggests that these compounds will be extremely useful tools in future studies designed to define the specific biological role of this receptor.

To demonstrate the usefulness of compounds **1** and **2** in this respect, we demonstrated that both compounds inhibit lipolysis in human-derived SW872 cells, whereas only compound **1** does in murine 3T3-L1 adipocytes. Previous studies have indicated that SCFAs inhibit lipolysis in adipocytes and that this occurs via a G_i/o_-dependent mechanism (9, 12). However, at least in SW872 cells, this was not the case because **C3** actually markedly enhanced lipolysis, although via a mechanism that did not involve FFA2 because this effect was not blocked by the hFFA2 antagonist CATPB. This observation indicates that **C3** has additional cellular targets in SW872 cells and that any effect on lipolysis it may have through FFA2 is effectively masked by these much larger “off-target” effects. Clearly, this highlights a major challenge to using only SCFA ligands to assess FFA2 function that can only be addressed through the development of more potent and selective ligands, such as compound **1**.

In the 3T3-L1 cells, it is interesting to note that the potency of both **C3** and **1** to inhibit lipolysis (3.24 ± 0.36 and 5.92 ± 0.43, respectively) was very similar to the values we measured at mFFA2 for these compounds in the [^35^S]GTPγS assay (3.57 ± 0.10 and 6.27 ± 0.13, respectively), a measure of G_i/o_-dependent signaling. We also noted that, although not quite reaching statistical significance, there was a trend toward **C3** producing a greater maximum inhibition of lipolysis than **1**. This could reflect either that **1** acts as a partial agonist relative to **C3** in this signaling response or that **C3** also has additional non-FFA2 targets in murine adipocytes that also modulate lipolysis. However, given that we observe little sign of partial agonism for **1** in other assays employing mFFA2, off-target effects of **C3** perhaps represent the more likely explanation. Although we were unable to detect mFFA3 mRNA in the differentiated 3T3-L1 model adipocytes, certain studies have reported FFA3 expression in adipocytes (5), whereas another noted altered adipocyte function in FFA3 knock-out mice (8). Indeed, although the expression of FFA3 in adipocytes remains controversial (4), it is at least conceivable that, despite a lack of detection of mRNA, low levels of mFFA3 protein may be expressed in these cells and could account for the greater efficacy of **C3** relative to **1**.

Another critical feature of compound **1** in addition to retaining activity at the rodent orthologs of FFA2 is that it is extremely selective for FFA2 over FFA3. This should allow for the delineation of FFA2-mediated responses in cells that co-express both receptors as we were able to do in demonstrating that FFA2, rather than FFA3, mediates GLP-1 release from STC-1 cells. The similar efficacy of **C3** and **1** in this assay suggests that despite expressing FFA3, GLP-1 secretion by these cells in response to **C3** is predominantly through FFA2, a conclusion consistent with previous work (29).

It is somewhat surprising that such substantial differences in activity were observed between **1** and **2** at the rodent orthologs of FFA2, given their structural similarity. Indeed, the primary structural difference between the two compounds (in addition to different positions for their chlorine substituents) is the presence of a phenyl substituent in **1**, which is cyclopentyl in **2**. Interestingly, closer examination of the docking of these compounds to our homology model of hFFA2 shows that this phenyl/cyclopentyl substituent is predicted to be in close proximity to Ser-86, the position we have found that when mutated to glycine (the amino acid present in rodent forms of FFA2) results in substantial loss of potency only for the cyclopentyl containing **2**. This appears to be consistent with this position being critical to the reduced potency of **2** at the rodent orthologs and perhaps suggests that replacing the serine residue with a smaller glycine residue affects the position and orientation of **2** in the binding cavity, resulting in less favorable contacts with other residues. It also must be noted that, given the marked variation in function of **1** and **2** at the rodent orthologs, future studies that further extend the structure-activity relationship of these compounds, particularly by modification of the phenyl/cyclopentyl substituent, may provide a good approach to identify compounds with even better activity at the rodent orthologs. Finally, an obvious corollary of these findings is that because only small chemical differences between **1** and **2** result in profoundly different functional consequences between species orthologs of FFA2, any future ligands in this series will also need to be assessed empirically across species before being considered as useful tool compounds.

Our observation that ECL2 contributes to both the binding and selectivity of compounds **1** and **2** is also of note. Interestingly, the structure of the extracellular loops, and in particular ECL2, varies greatly among the GPCRs for which crystal structures are available (30, 31). However, despite (or perhaps because of) this, there are several examples of GPCRs where ligand selectivity is defined at least in part by ECL2 (30, 31). It is, therefore, perhaps not surprising that mutation of a single residue in ECL2 of FFA2, Gln-148, to glutamate, as it is in FFA1 and FFA3, could result in loss of potency to these ligands, whereas the reciprocal mutation, at least in FFA1, would result in a gain in function. The specific mechanism underlying how this residue may dictate FFA2 *versus* FFA1 selectivity may also be of interest. Previous work has shown that an ionic lock between this position in FFA1 and one of two positively charged arginine residues within the fatty acid binding pocket of this receptor regulates constitutive activity of FFA1 (23). We have also recently shown that similar ionic locks involving additional positions within ECL2 regulate constitutive activity in human and rodent orthologs of FFA2 and FFA3 (11). Therefore, it is tempting to hypothesize that mutation of Gln-148 to glutamate in hFFA2 allows for the formation of an ionic lock at this position similar to that present in FFA1 and that this effectively closes off the binding pocket to **1** and **2**. By contrast, in FFA1, mutation of Glu-145 to glutamine would alleviate this ionic lock, thus allowing for increased binding by these ligands. To more directly test this, we also generated the Q148A mutant of hFFA2 and found that, unlike the Q148E mutant, the alanine mutant did not show reduced potency to **1** and **2**. This observation indicates that it is the gain of the negatively charged glutamate, and not the loss of glutamine that results in reduced potency to **1** and **2**, a finding that appears to support the ionic lock hypothesis.

It was interesting that **1** did possess some activity at wild type FFA1, as well as increased activity at the E145Q mutant. This is perhaps surprising, given that there is no overlap in the endogenous ligands activating these receptors, nor have any previously described synthetic ligands for FFA1 been found to have activity at FFA2 (20, 32–37). The fact that **1** does show some activity at FFA1 indicates that there is at least some similarity in the tertiary structure of the binding pockets of these receptors and perhaps indicates that counterscreening FFA1 agonists against FFA2 and FFA3 as well as for FFA4 and PPARγ activity should be more routinely carried out in the future. The observation that **1** was more active at both wild type and E145Q FFA1 than was **2** is also strikingly similar to the pattern of activity these compounds have at rodent FFA2. Because we have demonstrated that a glycine instead of serine at position 86^3.29^ accounts for this effect in rodent FFA2, we note that FFA1 possesses alanine at this 3.29 position, a residue that is also non-polar and smaller compared with the serine present in hFFA2. This may suggest that the preference for **1** over **2** in FFA1 is perhaps dictated by factors similar to those that dictate this same preference in rodent FFA2.

We have described the first potent and selective orthosteric agonists of FFA2 and defined the molecular basis for their interaction with the receptor. Our demonstration that at least one of these ligands can be used to delineate specific FFA2 function in murine cell systems highlights the importance these compounds may have, particularly in possible future, preclinical drug development and proof-of-principle studies at this receptor. FFA2 represents an interesting therapeutic target for the treatment of various metabolic and inflammatory conditions (28), for which development has been slowed primarily by a lack of reasonably potent and selective ligands (4). Therefore, the description of compound **1** as tool to study FFA2 function as well as our detailed examination of the molecular basis for its potency and selectivity, which should aid in future ligand development at these receptors, will be invaluable to exploring the potential of FFA2 as a therapeutic target.

## Footnotes

2 The abbreviations used are:

GPCR: G protein-coupled receptor
SCFA: short chain fatty acid
pERK: ERK phosphorylation
hFFA2 and -3: human FFA2 and -3, respectively
mFFA2 and -3: mouse FFA2 and -3, respectively
rFFA2: rat FFA2
4-CMTB: 4-chloro-α-(1-methylethyl)-*N*-2-thiazolylbenzeneacetamide
CATPB: (*S*)-3-(2-(3-chlorophenyl)acetamido)-4-(4-(trifluoromethyl)phenyl) butanoic acid
GLP-1: glucagon-like peptide-1
TUG-891: 3-(4-((4-fluoro-4-methyl-[1,1-biphenyl]-2-yl)methoxy)phenyl)propanoic acid
GTPγS: guanosine 5′-3-*O*-(thio)triphosphate
eYFP: enhanced yellow fluorescent protein
ECL2: extracellular loop 2
BRET: bioluminescence resonance energy transfer
HBSS: Hanks' balanced salt solution.

## References

[1] Itoh Y. Kawamata Y. Harada M. Kobayashi M. Fujii R. Fukusumi S. Ogi K. Hosoya M. Tanaka Y. Uejima H. Tanaka H. Maruyama M. Satoh R. Okubo S. Kizawa H. Komatsu H. Matsumura F. Noguchi Y. Shinohara T. Hinuma S. Fujisawa Y. Fujino M. (2003) Free fatty acids regulate insulin secretion from pancreatic β cells through GPR40. Nature 422, 173–176 12629551 10.1038/nature01478

[2] Hirasawa A. Tsumaya K. Awaji T. Katsuma S. Adachi T. Yamada M. Sugimoto Y. Miyazaki S. Tsujimoto G. (2005) Free fatty acids regulate gut incretin glucagon-like peptide-1 secretion through GPR120. Nat. Med. 11, 90–94 15619630 10.1038/nm1168

[3] Oh D. Y. Talukdar S. Bae E. J. Imamura T. Morinaga H. Fan W. Li P. Lu W. J. Watkins S. M. Olefsky J. M. (2010) GPR120 is an ω-3 fatty acid receptor mediating potent anti-inflammatory and insulin-sensitizing effects. Cell 142, 687–698 20813258 10.1016/j.cell.2010.07.041 PMC2956412

[4] Hudson B. D. Smith N. J. Milligan G. (2011) Experimental challenges to targeting poorly characterized GPCRs. Uncovering the therapeutic potential for free fatty acid receptors. Adv. Pharmacol. 62, 175–218 21907910 10.1016/B978-0-12-385952-5.00006-3

[5] Brown A. J. Goldsworthy S. M. Barnes A. A. Eilert M. M. Tcheang L. Daniels D. Muir A. I. Wigglesworth M. J. Kinghorn I. Fraser N. J. Pike N. B. Strum J. C. Steplewski K. M. Murdock P. R. Holder J. C. Marshall F. H. Szekeres P. G. Wilson S. Ignar D. M. Foord S. M. Wise A. Dowell S. J. (2003) The orphan G Protein-coupled Receptors GPR41 and GPR43 are activated by propionate and other short chain carboxylic acids. J. Biol. Chem. 278, 11312–11319 12496283 10.1074/jbc.M211609200

[6] Le Poul E. Loison C. Struyf S. Springael J. Y. Lannoy V. Decobecq M. E. Brezillon S. Dupriez V. Vassart G. Van Damme J. Parmentier M. Detheux M. (2003) Functional characterization of human receptors for Short Chain fatty acids and their role in polymorphonuclear cell activation. J. Biol. Chem. 278, 25481–25489 12711604 10.1074/jbc.M301403200

[7] Nilsson N. E. Kotarsky K. Owman C. Olde B. (2003) Identification of a free fatty acid receptor, FFA2R, expressed on leukocytes and activated by short-chain fatty acids. Biochem. Biophys. Res. Commun. 303, 1047–1052 12684041 10.1016/s0006-291x(03)00488-1

[8] Zaibi M. S. Stocker C. J. O'Dowd J. Davies A. Bellahcene M. Cawthorne M. A. Brown A. J. Smith D. M. Arch J. R. (2010) Roles of GPR41 and GPR43 in leptin secretory responses of murine adipocytes to short chain fatty acids. FEBS Lett. 584, 2381–2386 20399779 10.1016/j.febslet.2010.04.027

[9] Ge H. Li X. Weiszmann J. Wang P. Baribault H. Chen J. L. Tian H. Li Y. (2008) Activation of G protein-coupled receptor 43 in adipocytes leads to inhibition of lipolysis and suppression of plasma free fatty acids. Endocrinology 149, 4519–4526 18499755 10.1210/en.2008-0059

[10] Schmidt J. Smith N. J. Christiansen E. Tikhonova I. G. Grundmann M. Hudson B. D. Ward R. J. Drewke C. Milligan G. Kostenis E. Ulven T. (2011) Selective orthosteric free fatty acid receptor 2 (FFA2) agonists. Identification of the structural and chemical requirements for selective activation of FFA2 *versus* FFA3. J. Biol. Chem. 286, 10628–10640 21220428 10.1074/jbc.M110.210872 PMC3060514

[11] Hudson B. D. Tikhonova I. G. Pandey S. K. Ulven T. Milligan G. (2012) Extracellular ionic locks determine variation in constitutive activity and ligand potency between species orthologs of the free fatty acid receptors FFA2 and FFA3. J. Biol. Chem. 287, 41195–41209 23066016 10.1074/jbc.M112.396259 PMC3510819

[12] Lee T. Schwandner R. Swaminath G. Weiszmann J. Cardozo M. Greenberg J. Jaeckel P. Ge H. Wang Y. Jiao X. Liu J. Kayser F. Tian H. Li Y. (2008) Identification and functional characterization of allosteric agonists for the G protein-coupled receptor FFA2. Mol. Pharmacol. 74, 1599–1609 18818303 10.1124/mol.108.049536

[13] Smith N. J. Ward R. J. Stoddart L. A. Hudson B. D. Kostenis E. Ulven T. Morris J. C. Tränkle C. Tikhonova I. G. Adams D. R. Milligan G. (2011) Extracellular loop 2 of the free fatty acid receptor 2 mediates allosterism of a phenylacetamide ago-allosteric modulator. Mol. Pharmacol. 80, 163–173 21498659 10.1124/mol.110.070789 PMC3127537

[14] Vinolo M. A. Ferguson G. J. Kulkarni S. Damoulakis G. Anderson K. Bohlooly-Y. M. Stephens L. Hawkins P. T. Curi R. (2011) SCFAs induce mouse neutrophil chemotaxis through the GPR43 receptor. PLoS One 6, e21205 21698257 10.1371/journal.pone.0021205 PMC3115979

[15] Brantis C. E. Ooms F. Bernard J. (8 4, 2011) Novel amino acid derivatives and their use as GPR43 receptor modulators. International patent application WO/2011/092284

[16] Hoveyda H. Brantis C. E. Dutheuil G. Zoute L. Schils D. Bernard J. (6 17, 2010) Compounds, pharmaceutical composition and methods for use in treating metabolic disorders. International patent application WO 2010/066682

[17] Shimpukade B. Hudson B. D. Hovgaard C. K. Milligan G. Ulven T. (2012) Discovery of a potent and selective GPR120 agonist. J. Med. Chem. 55, 4511–4515 22519963 10.1021/jm300215x

[18] Stoddart L. A. Smith N. J. Jenkins L. Brown A. J. Milligan G. (2008) Conserved polar residues in transmembrane domains V, VI, and VII of free fatty acid receptor 2 and free fatty acid receptor 3 are required for the binding and function of short chain fatty acids. J. Biol. Chem. 283, 32913–32924 18801738 10.1074/jbc.M805601200

[19] Ballesteros J. A. Weinstein H. (1995) Integrated methods for modeling G-protein coupled receptors. Methods Neurosci. 25, 366–428

[20] Christiansen E. Urban C. Merten N. Liebscher K. Karlsen K. K. Hamacher A. Spinrath A. Bond A. D. Drewke C. Ullrich S. Kassack M. U. Kostenis E. Ulven T. (2008) Discovery of potent and selective agonists for the free fatty acid receptor 1 (FFA1/GPR40), a potential target for the treatment of type II diabetes. J. Med. Chem. 51, 7061–7064 18947221 10.1021/jm8010178

[21] Hudson B. D. Christiansen E. Tikhonova I. G. Grundmann M. Kostenis E. Adams D. R. Ulven T. Milligan G. (2012) Chemically engineering ligand selectivity at the free fatty acid receptor 2 based on pharmacological variation between species orthologs. FASEB J. 26, 4951–4965 22919070 10.1096/fj.12-213314 PMC3509056

[22] May L. T. Leach K. Sexton P. M. Christopoulos A. (2007) Allosteric modulation of G protein-coupled receptors. Annu. Rev. Pharmacol. Toxicol. 47, 1–51 17009927 10.1146/annurev.pharmtox.47.120505.105159

[23] Sum C. S. Tikhonova I. G. Costanzi S. Gershengorn M. C. (2009) Two arginine-glutamate ionic locks near the extracellular surface of FFAR1 gate receptor activation. J. Biol. Chem. 284, 3529–3536 19068482 10.1074/jbc.M806987200 PMC2635034

[24] Burt A. R. Carr I. C. Mullaney I. Anderson N. G. Milligan G. (1996) Agonist activation of p42 and p44 mitogen-activated protein kinases following expression of the mouse δ opioid receptor in Rat-1 fibroblasts. Effects of receptor expression levels and comparisons with G-protein activation. Biochem. J. 320, 227–235 8947492 10.1042/bj3200227 PMC1217922

[25] Waldecker M. Kautenburger T. Daumann H. Busch C. Schrenk D. (2008) Inhibition of histone-deacetylase activity by short-chain fatty acids and some polyphenol metabolites formed in the colon. J. Nutr. Biochem. 19, 587–593 18061431 10.1016/j.jnutbio.2007.08.002

[26] Maslowski K. M. Vieira A. T. Ng A. Kranich J. Sierro F. Yu D. Schilter H. C. Rolph M. S. Mackay F. Artis D. Xavier R. J. Teixeira M. M. Mackay C. R. (2009) Regulation of inflammatory responses by gut microbiota and chemoattractant receptor GPR43. Nature 461, 1282–1286 19865172 10.1038/nature08530 PMC3256734

[27] Sina C. Gavrilova O. Förster M. Till A. Derer S. Hildebrand F. Raabe B. Chalaris A. Scheller J. Rehmann A. Franke A. Ott S. Häsler R. Nikolaus S. Fölsch U. R. Rose-John S. Jiang H. P. Li J. Schreiber S. Rosenstiel P. (2009) Protein-coupled receptor 43 is essential for neutrophil recruitment during intestinal inflammation. J. Immunol. 183, 7514–7522 19917676 10.4049/jimmunol.0900063

[28] Ulven T. (2012) Short-chain free fatty acid receptors FFA2/GPR43 and FFA3/GPR41 as new potential therapeutic targets. Front. Endocrinol. 3, 111 10.3389/fendo.2012.00111 PMC3462324 23060857

[29] Tolhurst G. Heffron H. Lam Y. S. Parker H. E. Habib A. M. Diakogiannaki E. Cameron J. Grosse J. Reimann F. Gribble F. M. (2012) Short-chain fatty acids stimulate glucagon-like peptide-1 secretion via the G-protein-coupled receptor FFAR2. Diabetes 61, 364–371 22190648 10.2337/db11-1019 PMC3266401

[30] Peeters M. C. van Westen G. J. Li Q. Ijzerman A. P. (2011) Importance of the extracellular loops in G protein-coupled receptors for ligand recognition and receptor activation. Trends Pharmacol. Sci. 32, 35–42 21075459 10.1016/j.tips.2010.10.001

[31] Wheatley M. Wootten D. Conner M. T. Simms J. Kendrick R. Logan R. T. Poyner D. R. Barwell J. (2012) Lifting the lid on GPCRs. The role of extracellular loops. Br. J. Pharmacol. 165, 1688–1703 21864311 10.1111/j.1476-5381.2011.01629.x PMC3372823

[32] Briscoe C. P. Peat A. J. McKeown S. C. Corbett D. F. Goetz A. S. Littleton T. R. McCoy D. C. Kenakin T. P. Andrews J. L. Ammala C. Fornwald J. A. Ignar D. M. Jenkinson S. (2006) Pharmacolgical regulation of insulin secretion in MIN6 cells through the fatty acid receptor GPR40. Identification of agonist and antagonist small molecules. Br. J. Pharmacol. 148, 619–628 16702987 10.1038/sj.bjp.0706770 PMC1751878

[33] Negoro N. Sasaki S. Mikami S. Ito M. Suzuki M. Tsujihata Y. Ito R. Harada A. Takeuchi K. Suzuki N. Miyazaki J. Santou T. Odani T. Kanzaki N. Funami M. Tanaka T. Kogame A. Matsunaga S. Yasuma T. Momose Y. (2010) Discovery of TAK-875: A potent, selective and orally bioavailable GPR40 agonist. ACS Med. Chem. Lett. 1, 290–294 10.1021/ml1000855 PMC4007909 24900210

[34] Christiansen E. Due-Hansen M. E. Urban C. Grundmann M. Schröder R. Hudson B. D. Milligan G. Cawthorne M. A. Kostenis E. Kassack M. U. Ulven T. (2012) Free fatty acid receptor 1 (FFA1/GPR40) agonists. Mesylpropoxy appendage lowers lipophilicity and improves ADME properties. J. Med. Chem. 55, 6624–6628 22724451 10.1021/jm3002026

[35] Christiansen E. Urban C. Grundmann M. Due-Hansen M. E. Hagesaether E. Schmidt J. Pardo L. Ullrich S. Kostenis E. Kassack M. Ulven T. (2011) Identification of a potent and selective free fatty acid receptor 1 (FFA1/GPR40) agonist with favorable physicochemical and *in vitro* ADME properties. J. Med. Chem. 54, 6691–6703 21854074 10.1021/jm2005699

[36] Christiansen E. Due-Hansen M. E. Urban C. Merten N. Pfleiderer M. Karlsen K. K. Rasmussen S. S. Steensgaard M. Hamacher A. Schmidt J. Drewke C. Petersen R. K. Kristiansen K. Ullrich S. Kostenis E. Kassack M. U. Ulven. T. (2010) Structure-activity study of dihydrocinnamic acids and discovery of the potent FFA1 (GPR40) agonist TUG-469. ACS Med. Chem. Lett. 1, 345–349 10.1021/ml100106c PMC4007913 24900217

[37] Houze J. B. Zhu L. Sun Y. Akerman M. Qiu W. Zhang A. J. Sharma R. Schmitt M. Wang Y. Liu J. Liu J. Medina J. C. Reagan J. D. Luo J. Tonn G. Zhang J. Lu J. Y. Chen M. Lopez E. Nguyen K. Yang L. Tang L. Tian H. Shuttleworth S. J. Lin D. C. (2012) AMG 837. A potent, orally bioavailable GPR40 agonist. Bioorg. Med. Chem. Lett. 22, 1267–1270 22217876 10.1016/j.bmcl.2011.10.118

